# Views on Adolescents’ Mental Health in Sweden—A Qualitative Study among Different Professionals Working with Adolescents

**DOI:** 10.3390/ijerph182010694

**Published:** 2021-10-12

**Authors:** Linda Beckman, Lisa Hellström

**Affiliations:** 1 Department of Public Health Science, Karlstad University, 651 88 Karlstad, Sweden; 2 Department of School Development and Leadership, Malmö University, 211 19 Malmö, Sweden; lisa.hellstrom@mau.se

**Keywords:** adolescents, mental health, professionals, qualitative methods

## Abstract

Professionals who meet and work with adolescents hold important information and perspectives on adolescents’ mental health that, in addition to the adolescents’ voices, can shed light on complex issues. The aim was to explore professionals’ views on what challenges they face and how they can strengthen today’s adolescents’ mental health. This study involves four group interviews, conducted in March and October of 2020, including professionals with different working backgrounds. Data were analyzed with a qualitative content analysis. Two categories emerged: navigating life arenas and support for mental health. The first category included the demanding aspects of school, the challenges of social media, and the professionals’ thoughts on which pieces of mental health knowledge adolescents are lacking. The second category included what the professionals thought today’s parents need to develop and do to best support their children. Moreover, self-critical views were expressed on which aspects the professionals could do better to improve adolescents’ mental health. In conclusion, listening to professionals working with adolescents talk about adolescents’ mental health gives important insights. According to the professionals, both adolescents and their parents need improved life skills, including a strengthened and empowered self-esteem as well as improved mental health literacy.

## 1. Introduction

Self-reported mental health problems in children aged 10–17 have increased by over 100 percent in ten years in Sweden. For young adults, 18–24 years old, the increase is close to 70 percent. For psychosomatic symptoms such as difficulty sleeping, depression, irritability, nervousness, headaches, stomachaches, back pain, or dizziness, the proportion of 13- and 15-year-olds who report these disorders themselves has doubled since the 1980s [1]. Similar increases can be seen in other western countries [2,3]. However, self-reported mental health problems decrease during the transition to upper secondary school, after having increased steadily during adolescence compulsory school years [4]. It is also interesting to note that gender differences in the prevalence of mental health problems change during puberty. Before puberty, there is no significant difference between boys and girls reporting mental health problems [5], or boys might even report more behavioral problems [6]. However, after puberty, girls dominate the reports of increasing emotional problems [5].

Often, adolescents are unreflectively framed as a homogeneous group that increasingly suffers from mental health problems [7]. However, the picture of mental health among adolescents is ambiguous. Some arguments state that the increase is due to an increase in diagnoses rather than a true increase in incidence [8], or that people are more prone to share feelings, such as suicidal thoughts, due to better mental health literacy [9]. If the rise is real there are several explanatory models highlighted that can be linked both to the individual and to further structural factors, and mental health, e.g., unemployment [10,11], socio-economic preconditions [12], the psychosocial environment in school [13], and school performance [11,14,15], as well as gender norms and expectations [16]. Further, social media has been viewed as a source of mental health distress. Abi-Jaoude, Naylor, and Pignatiello [17] discussed that social media can affect adolescents’ self-image and interpersonal relationships through social comparisons and negative interactions; in the worst case, social media content might involve normalization and even promotion of self-harm and suicide among young people. Diagnosing mental health problems and illness based on self-reported symptoms also risks, as Wickström and Lindholm [18] argue, further neglecting social and structural determinants of health. In a qualitative study by Kvist Lindholm and Wickström [19], the authors found that young people may use psychiatric labels such as anxiety and depression as categories for everyday issues and strains in their lives, and these transformations and devaluations of these psychiatric categorizations might lead to misunderstanding the rise in self-reported mental ill-health. Further, the definitions of mental health problems have been widely debated since it can be difficult to distinguish between mental disorders and ordinary emotional expressions as they sometimes overlap [20]. In this study, our focus is not on specific mental disorders, but on professionals’ views of adolescents’ mental health.

The extent to which adolescents cope with everyday stressors is affected by a range of factors such as genes, disabilities, and life circumstances [21]. According to the World Health Organization [22], life skills are described as abilities for developing adaptive and positive behavior that enables individuals to cope with the demands and challenges of everyday life. Hence, there are innumerable life skills, and the nature and definition of life skills are likely to differ across cultures and settings. However, a core set of life skills has been suggested through health promotion research on children and adolescents. These are decision-making, problem-solving, creative thinking, critical thinking, effective communication, interpersonal relationship skills, self-awareness, empathy, coping with emotions, and stress. Specific emotional, cognitive, behavioral, and resilience skills play a vital part in adolescents’ personal and social success [22,23], and although there is some ambiguity in the results of life skills interventions [24], children and adolescents with psychosocial skills show more positive mental health and wellbeing [22]. There is a growing demand to educate adolescents about life skills, to help them deal with their day-to-day life challenges and transition into adulthood with informed healthy choices, but there are yet few qualitative studies on this subject [25]. Mental health literacy, a conceptual framework, offers ways of understanding and supporting mental health educational needs within the general population [26]. The concept of mental health literacy is defined as “understanding how to get and maintain positive mental health, understanding mental health problems and their treatments, decreasing stigma related to mental health problems, and enhancing help-seeking efficacy” [27].

Parents play an important role in the development of life skills in adolescence. Pathways to health, well-being, and positive social functioning have their roots in childhood, and perhaps the most powerful predictor of these pathways is the quality of early family and parenting environments to which the child is exposed. According to a review [28], parental involvement in the form of reading at home, holding high expectations/aspirations for their children’s academic achievement and schooling, good communication between parents and children regarding school and parental encouragement and support for learning correlated positively with academic success. Wang and Sheikh-Khalil [29] found that parental involvement improved academic and emotional functioning among adolescents. In addition, parental involvement predicted adolescent academic success and mental health both directly and indirectly through behavioral and emotional engagement.

The family home is thus a strategic setting for targeting prevention and early intervention for adolescents’ mental illness. Adolescents often turn to their parents if they have mental health difficulties [30,31] and most adolescents still live at home, which might help parents recognize significant changes in their child’s mental health and behavior [32]. Still, many adolescents with mental disorders do not receive professional help [33]. Barriers to help-seeking include a lack of knowledge about mental disorders and which professional help-seeking options are available [34], embarrassment or concern about what others might think, and a preference to seek help from family rather than professionals [32,34]. To provide this support, parents themselves need the relevant knowledge and skills [35]. However, surveys from several countries show that many adults also have limited mental health literacy in terms of prevention, recognition of when a disorder is developing, where to seek help, self-help strategies for milder problems, and first aid skills to support others affected by mental health problems [34].

In Sweden, work with adolescents’ mental health is conducted on many levels. There are clear arenas for this work (for example, school, healthcare, non-profit associations, organizations working with law and legislation). Still, many children and adolescents “fall between the cracks”, thus not receiving adequate help. According to a Swedish report [36], several Swedish regions report that their own organizations did not always know who was responsible for what when it comes to support and health care for adolescents with mental health problems. The first contact with health care for school-aged children in Sweden is primarily school health care. The school health care in Sweden is organized in so-called student health teams (SHT) and must, according to law, include the competence of a physician, school nurse, school social worker, and psychologist. Most schools also include remedial competence in this team. However, the Swedish SHT has been criticized by the OECD [37] and the Swedish School Inspectorate [38], pointing toward a sharp national understaffing. This critique resulted in a recent Swedish Government official investigation [39], which, among others, suggested introducing a numerical regulation of access to student health professionals.

Adolescents’ voices might differ from adults’ views on the same problem. Professionals who meet and work with adolescents most likely come in contact with adolescents’ mental health issues when it comes to treatment or being a significant adult. These professionals hold important information and perspectives on adolescents’ mental health that, besides what adolescents themselves can contribute with, can shed light on complex issues. Gathering professionals from different arenas can create a synergy effect of perspectives, which is useful for health-promoting work. Qualitative research studies on adolescents’ mental health are still scarce, and we need to put the numbers of mental ill-health in a narrative context. As far as we know, this study is the first in Sweden to gather professionals with different working backgrounds to talk about how they view this complex problem. Hence, in this study, we explored the challenges and how to strengthen adolescents’ mental health from the perspectives of professionals meeting adolescents in the course of their profession.

## 2. Materials and Methods

In this qualitative study with group interviews, we (L.B. and L.H., first and second author) conducted group interviews during March and October of 2020. This study is part of a larger project called Creating Better Life Skills among Adolescents. Besides group interviews with professionals, group interviews with young adults [40] have been conducted within the larger project. A previous research report with preliminary results from the study have been published [41].

### 2.1. Participants and Recruitment

The professionals (*n* = 10) were recruited via a Swedish national insurance company (Länsförsäkringar, LF) in three different Swedish cities. LF consists of 23 regional insurance companies that are located all over Sweden that are committed to community involvement and work with social sustainability. Regional companies that showed interest in the study were asked to recruit professionals within different professions who meet adolescents in their work to participate in the study. Each city’s quotations are named “A”, “B”, “C”, and “D” in the result section. The professionals in group “A” were represented by a child trauma professional, schoolteacher, and social worker. In group “B”, the professionals were represented by a coordinator for child psychiatry and social psychiatry, a coordinator on a primary health care unit for substance abuse and mental illness in children and a leisure center leader. In group “C”, the professionals were represented by a schoolteacher and a municipal project investigator for a project on mental health, and in group “D”, both professionals worked within the County council were one was a public health strategist and the other had an overarching focus on social services at a regional level. There were two groups from one of the cities. No particular selection criteria were stated other than that they should meet adolescents in their profession. The groups were put together based on which city they came from, and not on type of profession. 

The participants were given written information about the study in advance and that participation was voluntary; they could terminate their participation at any point. Informed consent was collected at the same time as the interviews.

### 2.2. Interview Guide and Procedure

The study was approved by the Swedish Ethical Review Authority (No: 2020-01600). The group discussions with professionals were conducted using a semi-structured interview guide consisting of a set of key questions. The interview guide was discussed with experts in the field prior to interviews. The main questions were: (1) According to your experience, what are the biggest challenges today’s school-aged children face in their everyday lives? (2) What do you think today’s school-aged children need to have the ability to cope with life’s successes and setbacks? (3) How do you work with adolescents’ mental health in your organization? (4) In what way do you collaborate with other actors and organizations regarding adolescents’ mental health? For each of the main questions, we had prepared some follow-up questions. Both authors were present at all interviews and took turns in leading the sessions. One group interview was conducted before the outbreak of the COVID-19 pandemic and the remaining interviews were conducted via the digital tool “Zoom”. Altogether, four group interviews (*n* = 3, *n* = 3, *n* = 2, *n* = 2) were conducted with the professionals and the interviews lasted between 60 and 80 min. The transcripts were not shared with the participants before or following the analysis.

### 2.3. Analysis

Data were transcribed verbatim in Swedish and analyzed using a qualitative content analysis, according to Granheim and Lundman [42]. It was later translated into English with the help of a native speaker. All texts from the group interviews were first read through to grasp the content. All interviews were analyzed simultaneously. First, the first author identified the units of meaning. These could be sentences, phrases, or words related to the aim of the study. Second, the meaning-bearing units were condensed and shortened without losing the essence of the message. Third, each condensed text section was labeled with a code representing its content, which could be discrete objects, events, or other phenomena, and the codes were interpreted in relation to the context. The codes were continuously compared to identify both differences and similarities. Fourth, the codes were divided into subcategories that expressed the latent content of the text. Fifth, subcategories formed two categories. During stages 4 and 5, the preliminary subcategories and categories were discussed with the second author.

### 2.4. Reflexivity and Research Team

The authors (L.H. and L.B., both women) have a Ph.D. in public health science where LB is an associate professor in public health science and LH is an associate professor in educational sciences, both with a specific research interest in mental health. Both authors have previous experiences in qualitative research. The current study is reported in line with the consolidated criteria for reporting qualitative research (COREQ) checklist [43].

## 3. Results

Two categories and five subcategories emerged from the analysis (Table 1): navigating life arenas and support for mental health. 

### 3.1. Navigating Life Arenas

This category included the subcategories school—a demanding arena, navigating social media, and knowledge of mental health. The category included the professional’s views on challenges in adolescents’ life and what they need to develop in order to handle life.

#### 3.1.1. School—A Demanding Arena

The school arena was seen, from the perspective of the professionals, as a challenge for adolescents, with too many demands for some children, especially for those who did not receive enough support from home. The way school is organized may not be favorable for everyone, and this was expressed to be even more obvious nowadays when education is in strong demand and a protective factor against exclusion. If you don’t complete high school, the future alternatives are few: *“And the demands at school and the performance are high, and it has always been that way for some… the current school form does not suit everyone, and so it has probably been also before but…”* (Group D).

Further, social media was described as a source of pressure even for academic success; the pressure about being successful meant that for many adolescents, it wasn’t enough to have just “pass” as a grade:

*“It is that they feel a lot of pressure. So, there are a lot of demands at school and you feel that there will be a lot… well you feel that you should succeed and, I do not know, we have of course discussed this a lot at work how it was before and how it is now, like with social media, and that it puts a lot of pressure on them to become… if you make sure to succeed, it is not enough to just be. Or it is not enough to just pass a subject, you have to be the best. And that can put a lot of pressure on many people. Some can do it, or many can do it. Most people can do it, but those who don’t… it will be a difficult thing.”* (Group B).

According to the professionals, adolescents today are given so many choices and it can be hard to navigate through all of these. These opportunities can be very stressful. Some navigate this easily, but for some, it can be very difficult. According to the professionals, these challenges should also be viewed as a structural problem. Background and family situations have a great impact on adolescents’ lives, and as with everything else, individuals exposed to more risk factors such as socioeconomic disadvantages, are at greater risk of being affected more negatively, especially within the school arena.

#### 3.1.2. Navigating Social Media

The impact of social media was raised by the professionals in several respects, contributing to a skewed picture of reality. Many school-aged children have a hard time navigating among all the inputs of social media, which can include abuse and destructive films and pictures, and attach great importance to the attention being given via likes on personal posts. The role of famous influential people online, i.e., “influencers”, came up and it was discussed how great of an impact they have on today’s school-aged children:

*“But then there are also a lot of famous YouTubers and people like that, that post their storytimes, as they call it, about their mental illness where they describe very serious problems. And so these 10-year-olds see this and think “God, I also have a stomach ache sometimes and I may have ADHD and I will end up in a foster family”. So I’ve heard a lot of kids talk like that because they kind of have an extreme version of it. So then they think that when their feelings arise that this is “I have the same as this or that famous person”…”* (Group A).

Hence, the discrepancy between social media and the real world was seen as too wide for adolescents to handle alone, and that they may need to learn more clearly that life is not all glamour. The constant comparison with people on social media was explained as the primary reason for this impoverishment of their self-image. Further, social media was seen to convey a picture of total happiness where no one ever makes any mistakes:

*“So both this with your self-esteem and self-confidence, to find yourself, to have strength, to dare to be who you are based on the requirements that I think are quite a lot for many children and young people. Then I also think of… in the big picture, what requirements does society set for you to… it really covers everything, social media, newspapers, television, the press on how to be. And then you also have to consider school and the performance there.”* (Group D).

#### 3.1.3. Knowledge of Mental Health

According to the professionals, adolescents are being fed with the notion that being sad is something dangerous, while it is just a normal reaction to life. The professionals expressed that school-aged children today seem to have low self-esteem, a weak self-image, and self-efficacy:

*“… you have such self-contempt. So build self-esteem, this with self-confidence… The whole bit I think is very important and like… well, there are so many that are very fragile. Boys too, but most of all right now I notice that there are very many girls who feel very mentally ill. And often you beat yourself down and it comes from all the demands and such that you do not think you are worth anything.”* (Group B).

The experience was that adolescents today need to have knowledge about what mental health can be, to be able to handle it adequately. According to the professionals, adolescents need knowledge about what anxiety is, what OCD (obsessive-compulsive disorder) is, when a condition is “normal”, and when professional assistance is required. According to the professionals, adolescents need help developing strategies to use when they are feeling down; ranging from staying home to rest, to seeking help if needed. Such strategies could help school-aged children to help their friends—which also emerged as a weight on the young. However, adolescents also needed to, most simply, be aware that it is okay to be sad, life is not perfect; this is something adolescents today need help to realize:

*“We do not equip children and young people with what life is like, I think… you do not really know what ill-health is, and what life is. Life goes up and down, and my experience is that not everyone knows that. So when you experience setbacks, you think you have mental illness, that is, that something is wrong, do you understand what I mean?”* (Group B).

The professionals discussed that there is a societal trend in medicalizing normal feelings, and this needs to be questioned by the adolescents themselves. Many adolescents today think they are the only ones who are experiencing such strong feelings, thinking they have a mental illness when feeling chest pressure before a big test or speaking in front of the class, while these are only normal feelings.

They further discussed that school-aged children today have a “fragile shell”. Today we talk much about feelings—for better or worse—but back in the days you just went out and “met” life. One participant said:

*“But I think that with many who come to me spontaneously, it is often about them presenting problems that are actually a part of life… I hear of course very, very serious problems also where you need to be referred further to adequate and professional help. But there are also many who are a little afraid to go out and face life. It is a challenge to deal with things that you as an adult may see as a part of life and that is, if you are to speak metaphorically, where I experience that very, very many have a fragile shell. They are not well equipped.”* (Group A).

However, another professional thought somewhat differently:

*“...if this is something that is in the spotlight right now with mental illness… you do not know… I can go back to 10 years ago when I started at the youth center… It feels like the adolescents then coped with adversity better, but you do not know if it was maybe that they went home and felt much worse and that now you talk about it, and now we see this as a huge problem. Probably it has always been there, but it has not been raised and I think we talk about much more today”* (Group A).

### 3.2. Support for Mental Health

The category support for mental health included the subcategories parental engagement, need for accessibility and self-critical eyes. This category included what the professionals thought about students’ health team (SHT) and what they thought today’s parents needed to develop and do to best support their children. Moreover, some self-critical eyes were turned to the professionals themselves concerning what they could do to better succeed in increasing adolescents’ mental health.

#### 3.2.1. Need for Accessibility

The need for accessibility, viewed from the professionals’ perspective, included aspects of how, primarily the students’ health team (SHT), including school social workers, school nurses and psychologists, and sometimes remedial competences, could be more accessible and available for the adolescents. They wished for the possibility for school social workers to be closer to the students, meet them outside on the schoolyard—not only when they had set up a meeting. It was expressed that the traditional structure of the SHT did not match the students’ preferences. There was some critique about the office hours, which in some schools could be limited to one hour per day when students can visit the SHT discretely:

*“Where are the adults in the school? It is sensitive to show that you (the adolescent) have to go to the school social worker, that everyone will see that you do it. The school social worker should be much more available and visible and be a part of the school. Be visible on breaks between classes, be available in a different way, join lectures, and so on, so that it becomes more natural to go to the school social worker.”* (Group D).

It emerged from the interviews that the support function often was person-dependent, meaning that how the person intended to support the adolescent and approached them was more important than the function itself. One participant said, *“Unfortunately you should be lucky when it comes to which adult kids meet in school, unfortunately, they are very person-dependent”* (Group C).

The important thing was that the adults were safe, present, and accessible.

#### 3.2.2. Parental Engagement

The professionals’ experiences of meeting with parents were that many times adults are poorly acquainted with their children’s interests, in particular with social media, and they do not keep up with new things, e.g., new apps or new platforms, leaving their children all alone with understanding and interpreting all the information produced. Adults are interpreted as lacking insight into what is happening on social media. One participant said: *“So many adults do not see that this is real-life for these adolescents. So they (adolescents) are left alone with it, and it becomes a bit closed from adults’ insight into what is actually happening there.”* (Group A).

It also emerged that the participants believed that parents needed more knowledge on what mental health is, when it is considered a problem, and how to seek help. This supportive education should start early and primarily include strengthening the parental role since there was an opinion that the parental role has been impoverished and that parents, in general, did not know how, or which limits they should set for their children. The professionals expressed that parents today seem to be afraid of being met with resistance and strong reactions from their children. There is, according to the professionals, an idea that it is dangerous (for the adolescent) to be disappointed, and above all that it is harmful to feel discomfort and to be nervous. One participant said:

*“Parents usually almost panic “Oh, my child is feeling really bad right now, he has said that he wants to die when he has a test tomorrow”. As if it were a suicide risk assessment when it’s really just… particularly adequate ordinary anxiety, but there is so much knowledge lacking in children and adolescents, and parents. So, you would like education for both parents, expectant parents, and for the children themselves.”* (Group A).

Another aspect that was expressed by the professionals was that the parents many times may be overreacting to their child’s feelings and reactions. One professional said that parents today build gated communities around their children. This could be explained, according to the professionals, by the idea that because parents today constantly hear and read about mental illness and self-harming behavior, they become scared of missing a sign of self-destruction in their own child.

There was a view that parents today need to better prepare their children for life and what comes with it, including tough times. They encouraged parents not to be afraid of difficult conversations with, or strong feelings expressed by, their child, and to encourage their children to challenge themselves in situations that could lead to anxiety or worry, such as having presentations in front of the class which could be a real trigger for anxiety. Adults need not underestimate their children’s abilities and instead let their children experience adversity, learn from setbacks, and not be afraid of difficult emotions, according to the professionals. Parents, the adults, need to be “square” and nuance children’s perspectives. One participant said: *“And then you have to prepare them for the fact that life will be difficult at times and then you have to be able to handle it without taking any drugs”* (Group B).

Parental support was viewed as fundamental for children’s health and there was a strong wish for them to be strengthened in their role as parents. However, it was also raised that this—reaching out to parents—was a great challenge as well, since in all preventive work, those who need support the most are the ones who are the most difficult to reach out to.

#### 3.2.3. Self-Critical Eyes

Collaboration, consensus, and co-action were seen as very important for the professionals’ work to meet the needs of adolescents, but that this was something they often failed succeeding in. Self-critical eyes were turned to themselves:

*“Are we doing the right things?… But somehow if you still have to put it in perspective, that it is actually the case that self-reported mental illness is increasing, then somewhere my conclusion is that we are not doing the right things. So, we obviously do not do enough… and if you come back to this with participation and what adolescents say, then maybe it is also the case that we have only now started to get a little better at what children and adolescents actually want and what they think of this. What kind of help do they want ?!”* (Group C).

Challenges in their work were about lack of time and knowledge from management and high costs for implementing collaboration and health promotion work. The efforts were often too short and lacked systematic sustainability to produce effect. One consensus they had was that the adolescents must be co-creative in the development work. Another perspective that came from the health care system was that you need to become much better at seeing adolescents as close relatives. Many have contact with the health service due to addiction problems. If they had received the right support much earlier, there would have been a difference, some argued.

## 4. Discussion

### 4.1. Results Discussion

This study explored the challenges and how to strengthen adolescents’ mental health from the perspectives of professionals meeting adolescents in the course of their profession. First, it is imperative to emphasize that these perspectives may or may not be shared by adolescents and may not reflect a true picture of reality. However, listening to the voices of adults meeting adolescents in different professional roles contribute to the current state-of-the-art research concerning adolescents’ mental health and how health promotive work can be strengthened. Hence, professionals’ perspectives should be seen as complements to adolescents’ voices. To understandpara how adults can meet the needs of adolescents, listening to professionals’ perspectives and hearing their voices may be an important first step. Navigating life arenas and support for mental health emerged as categories regarding professionals’ views on what life skills today’s adolescents need and what they are being challenged by when it comes to mental health.

In our study, challenges that were expressed by the professionals as a source of stress mainly circled around expectations at school and social media. The professionals experienced that adolescents lacked self-esteem, that they thought they needed to be perfect and that they constantly compared themselves to others on social media. This was seen as a problem across both genders but might have been more pronounced among girls. According to Odenbring [16], girls might have more demands to live up to, compared to boys, including academic success, being active on social media, heterosexual ideals of being good-looking, as well as being a good friend and daughter. However, the results also showed that the professionals expressed worry over adolescents’ lack of strategies to handle the discrepancy between social media and the real world. Similar results were reported in a qualitative study by Johnsen Hjetland et al. [44] among teachers talking about their experiences with adolescents’ social media use. They discuss that realistic life situations with challenges and hardship are not often displayed on social media, resulting in skewed comparisons to others’ seemingly perfect lives. This was, according to the teachers, a contributing factor to the impaired mental health among adolescents. Twenge [45] discusses that adolescents today experience a disconnection between expectations and reality—they are told “you can be anything you want to be’’ and ‘‘you are special’’ and then find out that reality is not so easy—but that this reflects their culture, and adolescents are forming their understanding of the world based on what their parents, teachers, and the media have taught them.

The professionals further expressed that adolescents were challenged by a lack of support functions or access to them. It was expressed that the traditional structure, including availability, of the SHT (student’s health team) was not perceived to match the students’ preferences. According to the professionals, students today want to have a more accessible SHT with more office hours. Maybe this could be solved with digital solutions, as this has already been tested in some Swedish schools [46] and elsewhere [47] during the COVID-19 pandemic with positive experiences. However, those working in the SHT might also feel overwhelmed by their situation, and in a review by Ravenna and Cleaver [48], school nurses (one of the common positions in the SHT) experienced several barriers to working with adolescents with mental health problems in schools. The main aspects included the time-consuming nature of caring for adolescents with mental health problems, large caseloads, and lack of training in mental health. However, in Sweden and other Western countries, the expansion of easily accessible “first line” centers aiming at helping troubled adolescents has been extensive, yet this issue still remains. In a parallel study where we talked to young adults [40], the participants said that it was the person rather than the function that mattered for positive help-seeking behavior. Similarly, the professionals in our study said that such functions are very “person-dependent”. The answer may then be available: trusting adults rather than additional professionals.

Parental engagement and support were the most important aspects discussed by the professionals in our study. It is well established that parental engagement and support in children’s lives affect their wellbeing in many ways [28,29,49]. For parents to provide support for their children, who may have temporary or chronic mental health problems, parents themselves need relevant knowledge and skills [35]. However, there is evidence that many adults have limited mental health literacy, which in turn reduces their ability to provide support [34,50]. This was confirmed from the perspectives of the professionals in our study. Lack of knowledge might increase the risk of assessing a child’s situation and wellbeing in an unreasonable way or even on the edge of suicide. In line with the views of the professionals in this study, it is preferable that parents also join in a nuanced debate of the mental health crisis where normal life experiences are not medicalized [19,51]. However, there is a kind of paradox in the relationship between today’s children and their parents; as mentioned, the professionals experienced that parents today are much closer to their children, making them overreacting to normal feelings and too hastily interpreting these feelings as harmful. As shown in Kvist Lindholm and Wickström [19], adolescents use psychiatric labels such as anxiety and depression as categories for everyday issues and strains in their lives, which could contribute to adults and professionals interpreting adolescents’ mental health incorrectly. Paradoxically, the participants in our study expressed that parents should aim to be more involved and more present in their children’s lives. The most prominent example was about social media. Social media has a tendency to be blamed as a cause for mental health problems, although the evidence is ambivalent [52]. Social media can help adolescents and parents to increase their mental health literacy in terms of providing them with valuable sources of information and tips for self-support [17,52]. On the other hand, social media could potentially be harmful to adolescents in terms of increased risk-taking behaviors, cyberbullying, and negative influences on health and wellbeing, including reduced self-image and self-esteem [53]. One conclusion here is that we need to find ways for parents and adolescents to meet and learn from each other; the adolescent knows social media, but an older adult might know strategies to deal with situations.

The professionals witnessed or thought they had seen that the type of mental health problems that adolescents seem to experience today might have changed, with an increase in adolescents experiencing “milder” mental health problems. Many theories are explaining the contradiction as well as confirming it [8]. Although it was expressed that adolescents need a stronger shell, we should be careful to interpret it as today’s adolescents needing to “toughen up”. Instead, “stronger shells” may be manifested as tools and coping strategies. One strategy, among others, as outlined by our professionals, might be educating and thus increasing mental health literacy among adolescents. This work is already ongoing worldwide, and one example is the intervention Mental Health First Aid (MHFA) [54] whose primary aim is to increase mental health literacy among both adults and adolescents.

Although there might be particular life skills or coping strategies that may need to be improved among today’s adolescents [24], structural hindrances such as inequality, poverty, and discrimination, that are correlated bidirectionally with mental health [55,56], are important to consider. Interventions that empower individuals in vulnerable situations and structural problems going beyond the focus of mental health must take a greater place in prevention strategies [57]. One component of this is a collaboration between different actors that come in contact with adolescents. The professionals also mentioned that we need to involve the adolescents themselves in our strivering for well-being. This is certainly an important aspect, since adults do not have all the answers, and they too need tools and self-esteem to guide and support their children. The professional did lift some self-critical eyes on themselves asking: how can we work even better together on this issue, and are we doing the right things when self-reported mental problems still rise?

### 4.2. Strengths and Limitations

The strength of this study was in the diversity of professionals that agreed to talk about this topic. Group interviews were used to collect data which enriched the study. When expressing an opinion in a group, the other participants could show whether they agreed or not, which showed the significance of what was being said. This was taken into consideration in the analysis, where the statements that the participants in the groups agreed on were considered more important [58,59]. However, there was also a risk of some issues being “taboo” in a group discussion, and therefore not being discussed as openly compared to an individual interview. As the participants in our study were adults talking from a professional perspective, we believe that they did not exclude any particular opinions, subjects or experiences. Another risk was that the professionals were affected by the current mental health debate, and thus said things that were in line with public opinion rather than their own experiences. Another aspect was that the groups were put together based on which city they came from, and not based on profession. This was decided based on the original idea of conducting face-to-face interviews. Having the same type of professionals in one group might have yielded slightly different discussions, perhaps less diverse discussions due to similar pre-understandings and experiences of their work. Furthermore, the groups were rather small, which might have further limited the opportunities to get diverse opinions. However, we do believe that this way of mixing groups resulted in discussions that were more dynamic. Another limitation of this study might have been that we had to do the interviews by the digital tool Zoom. Not having the chance to meet the participants in person might have made them more restrained. However, for some, it might have been more comfortable without personal meetings as using the telephone or other techniques such as Zoom might increase feelings of anonymity, making respondents more relaxed and open, which in turn can decrease interviewer effects [60]. Moreover, Zoom could have been experienced as a flexible tool, giving more professionals the opportunity to take part in the study. The reason to use Zoom were the onset of the COVID-19 pandemic and while three out of four interviews were done during the pandemic, the discussions might have been affected by situations caused by work and mental burdens of the following restrictions.

## 5. Conclusions

Listening to professionals that work with adolescents talk about young peoples’ mental health gives important insights, but these perspectives need to be combined with the voices of adolescents to get a more comprehensive picture. Overall, we found that the professionals in the current study believe that adolescents—and the adolescents’ parents as well—need improved life skills, including a strengthened and empowered self-esteem as well as improved mental health literacy. In addition to the home environment, school might be the most important arena to work with these questions, where both teachers and the SHT play important roles. There is a challenge, however, in changing old structures to meet adolescents’ needs with scarce resources.

## Figures and Tables

**Table 1:** Showing the results of categories and subcategories.

**Sub** **categories**	**Categories**	
	**Navigating Life Arenas**	**Support for Mental Health**
	School—a demanding arena	Need for accessibility
	Navigating social media	Parental engagement
	Knowledge of mental health	Self-critical eyes

## Data Availability

The data presented in this study are available on request from the corresponding author. The data are not publicly available due to ethical reasons.
